# Clinical applications, advances, and future directions in hysterosalpingography

**DOI:** 10.3389/fmed.2025.1537506

**Published:** 2025-04-25

**Authors:** Ting Wang, Tiantian Dong, Fang Nie

**Affiliations:** ^1^Ultrasound Medical Center, Lanzhou University Second Hospital, Lanzhou, China; ^2^Gansu Province Clinical Research Center for Ultrasonography, Lanzhou, China; ^3^Gansu Province Medical Engineering Research Center for Intelligence Ultrasound, Lanzhou, China

**Keywords:** hysterosalpingography, contrast-enhanced ultrasound, transvaginal hydrolaparoscopy, infertility, fallopian tube patency

## Abstract

In recent years, the incidence of infertility has been on the rise, and accurately evaluating the tubal status is of great significance in the diagnostic work-up of infertile women. HyCoSy has a certain proportion of false positives and false negatives in the evaluation of fallopian tube patency. The new third-generation drug-or gene-loaded microbubble contrast agents will largely achieve the dual purpose of diagnosis and treatment in clinical application, especially in transvaginal four-dimensional hysterosalpingo-contrast sonography (TVS 4D-HyCoSy), which has significant clinical value in assessing tubal patency and perifallopian tube adhesions. This study mainly discusses the selection of current diagnostic methods for tubal infertility, the technical challenges, and suggestions for ultrasonic diagnosis and postoperative treatment.

## 1 Introduction

In recent years, the incidence of infertility has been on the rise, affecting 8–17% of women of reproductive age, with tubal infertility accounting for ~12.8% (1). Medical histories such as pelvic inflammation, pelvic tuberculosis, septic abortion, uterine cavity malformation, intrauterine device use, appendiceal perforation, or ectopic pregnancy may contribute to tubal injury (2–4). Accurate evaluation of tubal status is of great significance in the diagnostic work-up of infertile women. Currently, the commonly used methods for evaluating the fallopian tubes include X-ray hysterosalpingography (HSG), laparoscopy with chromotubation (LC), two-dimensional (2D) hysterosalpingo-contrast sonography (HyCoSy), and three-dimensional (3D) HyCoSy.

## 2 Current diagnostic method options

### 2.1 X-ray hysterosalpingography (HSG)

HSG is a traditional standard method for diagnosing female infertility related to pelvic disease. It can effectively detect potential pelvic factors of infertility, especially in the evaluation of fallopian tube patency and abnormalities in uterine cavity morphology and structure. In addition, HSG can indirectly assess the degree of fallopian tube peristalsis and pelvic conditions. It also plays a certain role in the treatment of infertility during the examination process. However, its false-positive results cannot be ignored. Furthermore, its use in clinical practice is decreasing due to the presence of ionizing radiation and contrast agent allergies.

### 2.2 Laparoscopy with chromotubation (LC)

LC is a method in which a catheter is placed in the uterine cavity, a laparoscopic probe is placed in the abdominal cavity through the navel, and methylene blue is injected into the uterine cavity catheter to achieve real-time visualization of the uterus and ovary (5). Previous studies have shown that, in infertility investigations, the laparoscope is superior to the hystero-salpingogram in diagnosing both the site and extent of tubal disease. LC has been recognized as the “gold standard” for evaluating fallopian tube patency in many settings, as it can directly detect the condition of the fallopian tube, perform intubation and dredging treatment of obstructions during the operation, and identify other pelvic lesions (6, 7). However, LC is invasive, requires hospitalization under general anesthesia, is expensive, and carries some risks of morbidity and even mortality. Therefore, it is generally not the first choice for evaluating tubal patency. In addition, studies have shown that endometrial cells in liquid chromogenic tubes can spread to the abdominal cavity, leading to an increased probability of endometriosis (8). A comparative analysis of 35 infertile patients showed that, using the laparoscopic staining method as a reference, the accuracy, positive predictive value, and negative predictive value of hysteroscopic tubal assessment were 82.9, 87.5, and 76.7%, respectively (9). However, this method has obvious limitations. Studies have shown that fuel in patients with adenomyosis will spread through the uterine wall and easily cause false-negative results in infertile women with obstructed fallopian tubes (10).

### 2.3 Transvaginal hydrolaparoscopy (THL)

THL is a modification of culdoscopy that can be used to evaluate the posterior uterus, pelvic sidewalls, and adnexae (11). Diagnostic THL can be performed in the office under local anesthesia. When combined with diagnostic hysteroscopy and chromotubation, it can replace HSG as the first-line diagnostic test for infertile women. Studies have shown high patient tolerability, with less pain reported post-procedure compared to HSG. THL has been shown to have a high concordance with HSG for tubal patency, but it also diagnosed more intrauterine abnormalities and identified adhesions and endometriosis that were not visible with HSG. In addition, salpingoscopy may be performed during THL to assess the tubal lumen. THL also has a high concordance rate with laparoscopy when a complete evaluation is accomplished during THL. Complications of THL are uncommon and typically minor. Finally, operative procedures such as ovarian drilling, coagulation of endometriosis, lysis of adhesions, treatment of ovarian cysts, and salpingostomy may be performed via THL.

### 2.4 Two-dimensional hysterosalpingo-contrast sonography (2D-HyCoSy)

At present, two-dimensional transvaginal ultrasound (2D-TVS) is the most commonly used method for diagnosing gynecological diseases in clinical practice. It offers advantages such as ease of use, high safety, non-invasiveness, and being painless. In addition, transvaginal scans do not require bladder filling, provide higher resolution images for more adequate diagnostic information, and are not affected by obesity and changes in the position of the pelvic organs (12). However, 2D-TVS only shows the longitudinal and transverse sections of the uterus and detects the presence or absence of abnormal hyperplasia. It cannot completely show uterine cavity adhesions and fallopian tube patency, making it prone to false negatives. Therefore, further evaluation is often needed with 2D-TVS HyCoSy (13), which is a method that combines contrast-enhanced ultrasound technology with two-dimensional ultrasound technology to evaluate tubal patency and diagnose uterine tubal patency (14).

There are two types of contrast agents commonly used in HyCoSy: negative and positive. The negative contrast agent is similar to a glucose or normal saline solution. After injection, the uterine cavity separates, dilates, and becomes anechoic, allowing abnormalities and lesions in the uterine cavity to be clearly visualized against the anechoic background (15). Previous studies have shown that saline hysterosalpingography is a reliable method for the diagnosis of uterine or fallopian tube disease in infertile patients (16).

In recent years, with the development of ultrasound medical technology, new positive contrast agents, such as hydrogen peroxide and other microbubble contrast agents, have been increasingly used in clinical practice (17). When the fallopian tube is filled with a contrast agent, it shows high enhancement, allowing for the accurate evaluation of fallopian tube patency. At present, a commonly used contrast agent is the macromolecular inert gas-based contrast agent, such as SonoVue (Sonovue). This type of contrast agent is a microbubble-based agent with a phospholipid-coated surface and sulfur hexafluoride gas inside. It is a safe, stable contrast agent that provides long-lasting performance. Therefore, 2D-HyCoSy is also becoming increasingly recognized in clinical practice (13, 18). Compared to X-ray HSG, 2D-HyCoSy has the advantages of being safer, faster, radiation-free, and having a lower incidence of allergic reactions (19). 2D-HyCoSy can also provide a very accurate assessment of the uterine cavity (20) and has high accuracy in assessing tubal patency in patients without endometriosis and those with endometriosis (21).

The study showed that the use of the SonoVue contrast agent in HyCoSy allows for easier evaluation of fallopian tube patency, a more accurate assessment of fallopian tube obstruction, and better visualization of the fallopian tube's course (22–24). 2D-HyCoSy has been shown to be as reliable as laparoscopic techniques in assessing tubal patency and uterine morphology, and it also overcomes major disadvantages such as the need for hospitalization, radiation exposure, anesthesia, and the use of iodinated contrast material. It is considered a safe and well-tolerated outpatient procedure, which clearly favors the occurrence of natural pregnancy (25).

However, traditional 2D-HyCoSy also has some limitations, which include the following: (1) the tortuous course of the fallopian tube, which limits the clarity of the scan; (2) the contrast agent overflowing from the fibrous end of the fallopian tube, making it difficult to distinguish from the intestinal tract surrounding the tube; and (3) the need for the entire procedure to be performed skillfully, accurately, and quickly, capturing all aspects of the uterus and fallopian tube in a short time for accurate diagnosis (26).

### 2.5 Three-dimensional hysterosalpingo-contrast sonography (3D-HyCoSy)

2D-HyCoSy cannot obtain multi-dimensional information, and its examination accuracy is relatively low. It cannot clearly and intuitively display uterine lesions, making missed diagnoses more common. Three-dimensional transvaginal sonography (3D-TVS) can capture any section according to specific needs, expand the observation range, and perform a three-dimensional reconstruction of the image. By using the spatial relationship, the shape of the uterine cavity can be clearly observed, and the image can be analyzed from multiple angles, thereby partially compensating for the shortcomings of two-dimensional transvaginal sonography.

The clinical application of 3D contrast-enhanced ultrasound (3D-CEUS) technology has provided more abundant and comprehensive imaging information for diagnosis. 3D imaging allows for better optimization of ultrasound contrast agents. It emits an ultrasound beam of a specific frequency through a three-dimensional probe and receives the contrast-enhanced ultrasound signal, which is characterized by a narrow beam. This process filters out the surrounding tissue signals, which are characterized by a wide beam, thus avoiding the superposition of tissue and contrast signals. As a result, the image only shows the contrast-enhanced ultrasound signal. This method, using low sound pressure, can develop the contrast agent in a very short time. Therefore, we can visualize the 3D shape of the uterine cavity and the fallopian tube (26). 3D-TVS HyCoSy is a procedure in which the contrast agent is pressurized into the uterine cavity through the vagina, and the coded CEUS imaging technique is used for 3D imaging of the uterine and fallopian tube lumen (27). It is an accurate method for diagnosing tubal occlusion in women with infertility (28).

In our previous studies, 126 patients (252 fallopian tubes) underwent 3D-TVS HyCoSy and real-time 2D-TVS HyCoSy examinations. According to the final 2D real-time evaluation, 111 patients had bilateral fallopian tube patency, four patients had bilateral fallopian tube obstruction, and 11 patients had unilateral fallopian tube patency. The conformity rate of fallopian tube patency status was as follows: the coincidence rate between the first 3D volume acquisition and 2D real-time evaluation was 84%, while the coincidence rate between the second 3D volume acquisition and 2D real-time evaluation was 97%. During the procedure, 58% of the patients' visual analog score (VAS) was > 5, and 85.7% of the patients' pain score was ≤ 5 at the end of the operation. We conclude that HyCoSy, using automatic analysis and 3D-HyCoSy technology, retains the advantages of conventional 2D-HyCoSy while overcoming the disadvantages. 2D-HyCoSy is highly observer-dependent and can only be performed accurately under the control of an experienced observer. In contrast, 3D volume acquisition enables the visualization of the fallopian tube in the coronal position by acquiring the volume data of the uterus and fallopian tube. This also allows the flow process within the fallopian tube to be observed in three-dimensional space, thus making it relatively easier for less experienced operators to evaluate fallopian tube patency.

Studies (29, 30) have shown that the sensitivity of combined two-dimensional and three-dimensional contrast-enhanced ultrasound in the diagnosis of adnexal lesions can reach 100%, but the specificity needs to be further improved. However, the acquisition of three-dimensional volume images requires close cooperation between the operator and the nurse to accurately determine the best scanning time and avoid losing information due to scanning too early or too late (31).

### 2.6 Four-dimensional hysterosalpingo-contrast sonography (4D-HyCoSy)

With the development of acoustic contrast technology and the introduction of ultrasound contrast agents, the diagnosis of hysterosalpingography has evolved from 2D-HyCoSy to 4D-HyCoSy. This advancement has increased diagnostic accuracy while reducing dependence on sonographers.

4D-HyCoSy overcomes the limitations of 2D-HyCoSy and demonstrates a relatively high degree of agreement with the more challenging 2D-HyCoSy technique (32). Studies have shown that, between transvaginal 4D-HyCoS and LC that are used in the diagnosis of uterine tubal patency, the former has greater clinical value in the evaluation of fallopian tube patency and adhesions around the fallopian tubes (33).

This team retrospectively analyzed the 4D hysterotubal contrast-enhanced ultrasound findings of 98 infertile women from 2017 to 2020 and conducted a follow-up analysis 6 months later to analyze factors affecting infertility. The results showed that the four-dimensional observation indices of tubal development, contrast agent overflow at the umbrella end, and the ring-like wrapping of the contrast agent around the ovary in diagnosing tubal patency were 100% consistent with laparoscopy. The delay in contrast agent extravasation time at the end of the fallopian tube is of great value in the evaluation of fallopian tube patency.

Studies have also shown that some infertile women can successfully conceive naturally after 4D-HyCoSy. Hence, 4D-HyCoSy is recommended as the preferred method for testing tubal patency, and infertile patients are advised to undergo the 4D-HyCoSy examination as early as possible (34).

Overall, HyCoSy had a sensitivity ranging from 76 to 96% for determining tubal patency, while its specificity ranged from 67 to 100% (6, 35, 36).

## 3 Technical challenges

### 3.1 False positives and false negatives

HyCosy has a certain proportion of false positives and false negatives in the evaluation of fallopian tube patency. Some uterine lesions, such as submucosal fibroids, adhesions, or abnormal uterine morphology, affect the filling and diffusion of contrast media, resulting in false-positive results. When the tip of the intrauterine catheter is close to one side of the uterine horn, it can affect the passage of the contrast agent, resulting in false positive signs of ipsilateral proximal fallopian tube obstruction. In addition, fallopian tube patency does not guarantee a 100% chance of pregnancy because abnormalities in oocyte pickup function or fertilized egg implantation may also affect the pregnancy rate (37–39). In such cases, it is necessary to distinguish between functional lesions and morphological lesions by doctors with rich clinical experience.

### 3.2 Complications and adverse reactions

Some patients have experienced pain or bleeding due to intrauterine adhesions or abnormal uterine morphology during catheterization or examination (40). Occasionally, patients have developed postoperative infections. In addition, contrast agent counterflow may happen during the examination. When the pressure in the uterine cavity is too high, the contrast agent can easily backflow to the myometrium, parametrial blood vessels, and lymphatic system, leading to the visualization of the uterine wall and parametrial tissue. Especially, when a large amount of lipiodol enters the venous system, serious adverse reactions may occur.

## 4 Future direction

### 4.1 Clinical application of new microbubble contrast agents

New third-generation drug- or gene-loaded microbubble contrast agents are expected to largely achieve the dual purpose of diagnosis and treatment in clinical application (41). Especially for conditions such as endometriosis (42, 43), inflammation, or tuberculosis caused by hysterofallopian tube lesions, the main feature of these agents is their ability to carry anti-inflammatory or anti-tuberculosis drugs or gene fragments on the contrast agent microbubbles. The contrast agent is injected until it reaches the target site of the lesion so as to achieve a new level of treatment effectiveness.

### 4.2 Four-dimensional hysterosalpingo-contrast sonography (4D-HyCoSy)

Transvaginal 4-dimensional hysterosalpingo-contrast sonography (TVS 4D-HyCoSy) is a highly useful method for diagnosing tubal patency. However, large-scale studies are warranted in the future to investigate our findings in patients with tubal infertility (44). Intravasation during transvaginal 4-dimensional hysterosalpingo-contrast sonography (TVS 4D-HyCoSy) may lead to false-negative results in the evaluation of tubal patency. The preoperative clinical and two-dimensional ultrasound features, along with the related medical history of patients (45), can be collected in advance to establish a prediction model for identifying the influencing factors of fallopian tube patency (46).

## 5 Recommendations for post-HyCoSy diagnosis and treatment

The methods of fallopian tube examination were compared in this study (Table 1). 4D-HyCoSy is suitable as an initial screening tool, especially for patients with radiation sensitivity or iodine allergy, and is moderately cost-effective. HSG is suitable for patients with limited financial means or those who require initial screening and mild treatment, but it is important to be aware of the risks of radiation and allergies. LC is suitable for complex cases, especially those requiring a clear diagnosis and simultaneous treatment, but it is expensive and invasive.

**Table 1:** Comparison of the advantages and disadvantages of tubal examination methods.

**Indicators**	**4D-HyCoSy**	**HSG**	**LC**
Diagnostic accuracy	High	Medium	Highest (gold standard)
Radiation exposure	No	Yes	No
Risk of allergy	Lower	Higher (iodolide contrast)	Lower
Aching sensation	Mild	Moderately	High pain (anesthesia required)
Cost	Medium	Lower	Higher
Therapeutic effect	No	Part (mild adhesion dredging)	Yes (can be operated at the same time)
Application scenario	Preliminary screening, radiation sensitivities	Limited finances, initial screening and treatment	Complex cases require clear diagnosis and treatment

Patients with bilateral patent fallopian tubes, as determined by ultrasound, are generally not treated. Expectant treatment for ~8–9 months is feasible for infertile women whose 4D-HyCoSy findings show unilateral tubal patency or poor patency (34). Obstruction of the proximal fallopian tube (interstitial part and isthmus) detected by 4D-HyCoSy requires directional intubation with hysteroscopy (47, 48), after which pregnancy attempts could be made the following month. Findings suggesting bilateral proximal tubal obstruction also require further evaluation to rule out the possibility of artifacts affecting the results due to transient tubal/myometrial contractions or issues related to the catheter position (49). However, for patients with a clear diagnosis of bilateral fallopian tube obstruction, an immediate clinical intervention is recommended (34).

Abnormal fallopian tube shape or distal (ampulla or fimbria) obstruction should be treated with hysteroscopy and laparoscopy (50–52). Individualized treatment is performed according to the patient's condition, such as releasing pelvic adhesion, restoring the normal shape of the fallopian tube, and dredging distal obstruction (53, 54).

Women with hydrosalpinx have lower implantation and pregnancy rates with assisted reproductive technology (ART), and current guidelines recommend removing the fallopian tubes through salpingectomy (preferably laparoscopic) prior to *in vitro* fertilization (IVF) treatment (55). Salpingostomy or distal salpingoplasty are alternative treatments for women with hydrosalpinx who wish to conceive naturally. However, the risk of ectopic pregnancy is higher after both salpingoplasty and fallopian tube replacement, especially following fallopian tube replacement (56). For patients with mild fallopian tube injury, salpingoplasty is the preferred option, but postoperative pregnancy needs to be closely monitored. If natural pregnancy is difficult after surgery or the risk of ectopic pregnancy is too high, it is recommended to consider assisted reproductive technology as soon as possible.

For patients with mild hydrosalpinx, it is recommended to perform open-end plasty or ostomy. For patients with severe hydrosalpinx that cannot be repaired surgically, laparoscopic salpingectomy is recommended before IVF-ET (57). If bilateral fallopian tube dredging treatment is performed, 4D-HyCoSy should be performed again the following month to determine the patency of the fallopian tube after treatment.

Overall, 4D-HyCoS and LC are very useful in the diagnosis of uterine tubal patency. The former has more clinical value in the evaluation of fallopian tube patency and adhesions around the fallopian tube. The specific choice of method should be weighed against the specific circumstances of the patient (such as financial conditions, complexity of the condition, and history of allergies) and the recommendations of the doctor.
